# Retraction: Aquaporin-4 Inhibition Mediates Piroxicam-Induced Neuroprotection against Focal Cerebral Ischemia/Reperfusion Injury in Rodents

**DOI:** 10.1371/journal.pone.0234828

**Published:** 2020-06-11

**Authors:** The PLOS ONE Editors

Following the publication of this article [**1**], concerns were raised regarding similarities in text and figures in this article and previously published articles in *Brain Research* [2, 3], *Neuropharmacology* [4], *Cellular and Molecular Neurobiology* [5], *Medical Hypotheses* [6] and *Anesthesia & Analgesia* [7]. Furthermore, the editors noted similarities between figures in this article and figures later published in *The Journal of Physiology and Biochemistry* [8] and *Life Sciences* [9]. Specifically:

- Within Fig 3A:
  ○ The first brain section in the Vehicle panel looks similar to the second brain section in the Vehicle + Drug (2 hr post) panel.
  ○ The second brain section in the Vehicle panel looks similar to the fourth brain section in the Vehicle panel.
  ○ The third brain section in the Vehicle panel looks similar to the fourth brain section in the Vehicle + Drug (4 hr post) panel.
  ○ The second brain section in the Vehicle + Drug (30 min pre) panel looks similar to the third brain section in the Vehicle + Drug (2 hr post) panel.
- The second brain section in the Vehicle + Drug 5mg panel of Fig 2A looks similar to the first brain section in the Vehicle + Drug (30 min pre) panel of Fig 3A.
- Similarities were also noted between images reported in this article and in other published articles [2–4, 8, 9]. In total, 19 out of 24 panels in Fig 2A and 11 out of 20 panels in Fig 3A appear similar to other images within this article and/or to images published in [2–4, 8, 9] that represent the results of different experiments.
- The following text excerpts overlap with text published in previous articles:
  ○ Materials and Methods section, subsections similar to [2] which was not cited as an original source for the procedures: “Evaluation of motor coordination and neurological scoring” (paragraph 2), “Estimation of cerebral infract volume and brain swelling”, “Biochemical Analysis”, “Measurement of Nitrite”, and “Estimation of Malondialdehyde (MDA)”.
  ○ Materials and Methods section, subsections similar to [5] which was not cited as an original source for the procedures: “Isolation of Total RNA”, “Reverse Transcription”, “Polymerase Chain Reaction”, “Tissue Lysate Preparation”, “SDS-Polyacrylamide Gel Electrophoresis”, and “Immunoblotting”.
  ○ Results section, subsections similar to [2]: “Dose optimization of Piroxicam”, “Effect of Piroxicam on pre and post treatment on cerebral infract volume and neurological deficit” (paragraph 1), “Effect of Piroxicam on brain nitrite levels”, and “Effect of Piroxicam on brain MDA levels”.
  ○ Discussion sections similar to [6]: Paragraphs 1, 2, 5, 6.
  ○ Discussion sections similar to [2]: Paragraphs 5, 6.
  ○ Discussion sections similar to [10]: Paragraphs 9, 10.
  ○ Discussion sections similar to [7]: Paragraphs 12, 13.

The authors stated that the similarities between panels within [**1**] and between panels in [**1**] and their own articles resulted from inadvertent mis-uploading of panels during figure preparation. They have offered replacement panels to address some of the issues outlined above. The authors also stated that they cannot comment on images published in different papers by different authors. The authors provided underlying data to support results in Figs 2A and 3A. However, the underlying data provided were not sufficient to resolve the concerns noted above, including the similarities between panels depicting different experimental conditions. Regarding text overlap, the authors stated that the similarity in methodology results from both labs using the same methods and that the overlap in the Results and Discussion sections was due to the authors’ unawareness of guidelines.

In light of the text overlap and the image concerns that call into question the reliability of the reported results and conclusions, the *PLOS ONE* Editors retract this article.

The panels of Figs 2A and 3A that are listed below appear visually similar to material published in 2010–2011 which are not offered under a CC-BY license. The indicated panels are therefore excluded from the *PLOS ONE* article's [**1**] license. At the time of retraction, the article [**1**] was republished to note this exclusion in the Figs 2 and 3 legends and the article’s copyright statement.

Visually similar to images in Figures 1C and 2C of [4], published in 2010 by Elsevier:

- The first and fourth brain sections in the Sham panel of Fig 2A
- The first and fourth brain sections in the Vehicle panel of Fig 2A
- The first, third, and fourth brain sections in the Vehicle + Drug 5mg panel of Fig 2A
- The first, third, and fourth brain sections in the Vehicle + Drug 10mg panel of Fig 2A
- The third and fourth brain sections in the Vehicle + Drug 20mg panel of Fig 2A

Visually similar to images in Figure 1 of [2], published in 2011 by Elsevier:

- The second, third, and fourth brain sections in the Vehicle panel of Fig 3A
- The fourth brain section in the Vehicle + Drug (2 hr post) panel of Fig 3A
- The third and fourth brain sections in the Vehicle + Drug (4 hr post) panel of Fig 3A

Visually similar to images in Figures 2C, 3C, and 4C of [3] published in 2011 by Elsevier:

- The second brain section in the Sham panel of Fig 3A
- The third brain section in the Vehicle + Drug (30 min pre) panel of Fig 3A

The authors did not agree with retraction.
